# Supplementary material for: DeepProg: an ensemble of deep-learning and machine-learning models for prognosis prediction using multi-omics data
Source: Genome Med. 2021 Jul 14;13:112. doi: 10.1186/s13073-021-00930-x (PMC8281595; doi:10.1186/s13073-021-00930-x)

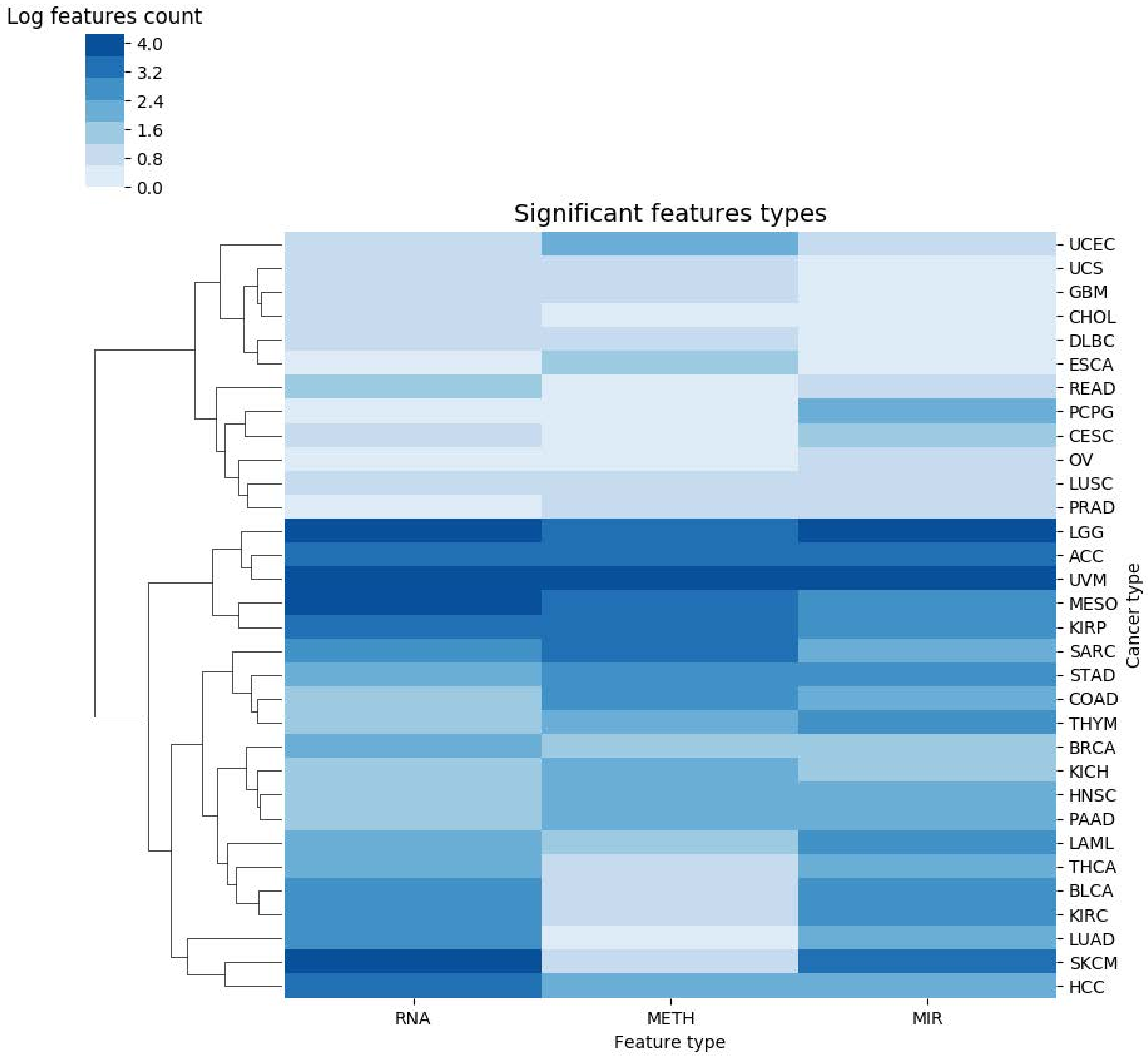

Log features count
4.0
3.2
2.4
1.6
0.8
0.0
Significant features types
UCEC
UCS
GBM
CHOL
DLBC
ESCA
READ
PCPG
CESC
OV
LUSC
PRAD
LGG
ACC
UVM
MESO
KIRP
SARC
STAD
COAD
THYM
BRCA
KICH
HNSC
PAAD
LAML
THCA
BLCA
KIRC
LUAD
SKCM
HCC
Cancer type
RNA
METH
MIR
Feature type

Figure S1

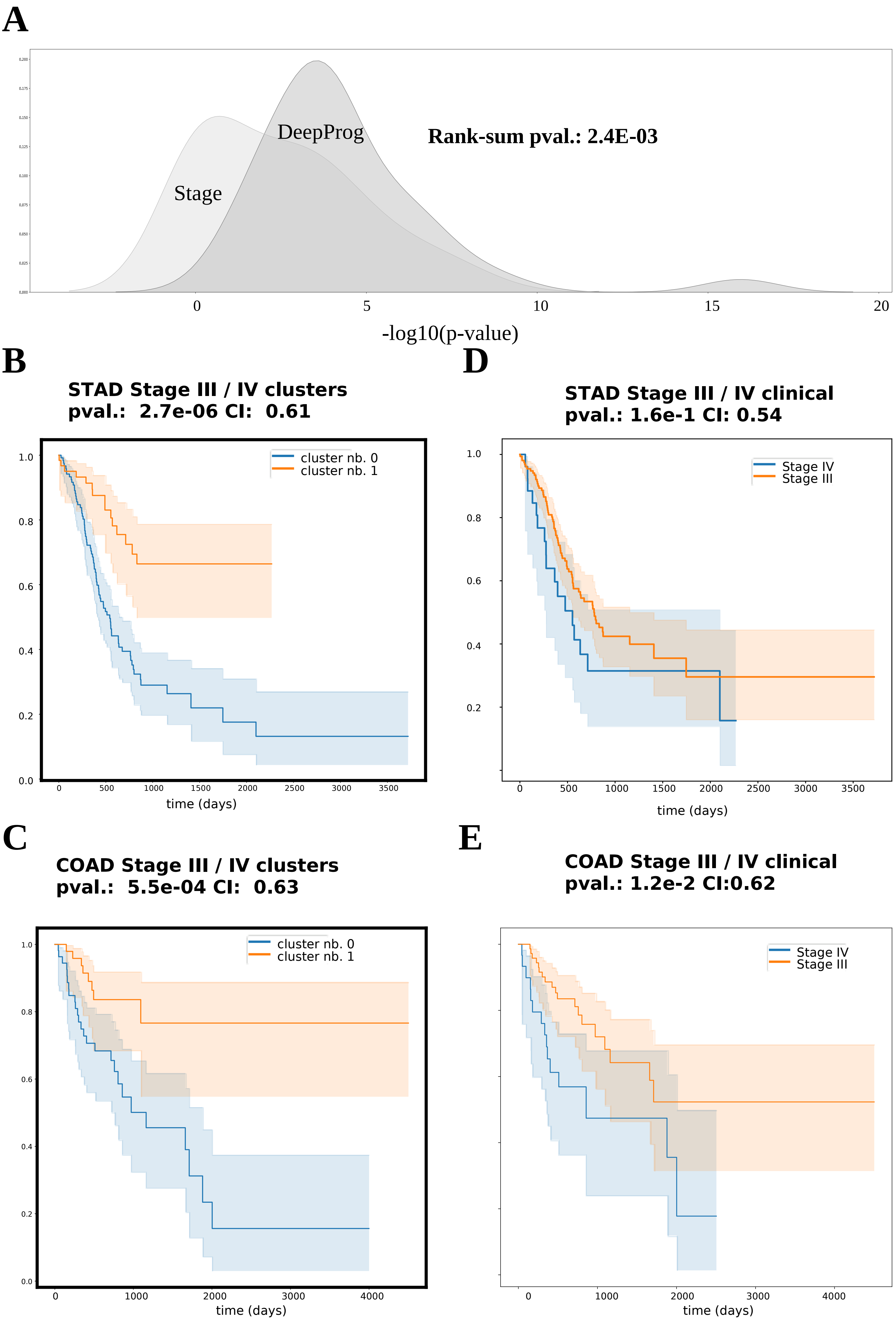
A
Stage
DeepProg
Rank-sum pval.: 2.4E-03
-log10(p-value)
B
STAD Stage III / IV clusters
pval.: 2.7e-06 CI: 0.61
cluster nb. 0
cluster nb. 1
time (days)
D
STAD Stage III / IV clinical
pval.: 1.6e-1 CI: 0.54
Stage IV
Stage III
time (days)
C
COAD Stage III / IV clusters
pval.: 5.5e-04 CI: 0.63
cluster nb. 0
cluster nb. 1
time (days)
E
COAD Stage III / IV clinical
pval.: 1.2e-2 CI:0.62
Stage IV
Stage III
time (days)

Figure S2

Figure S3

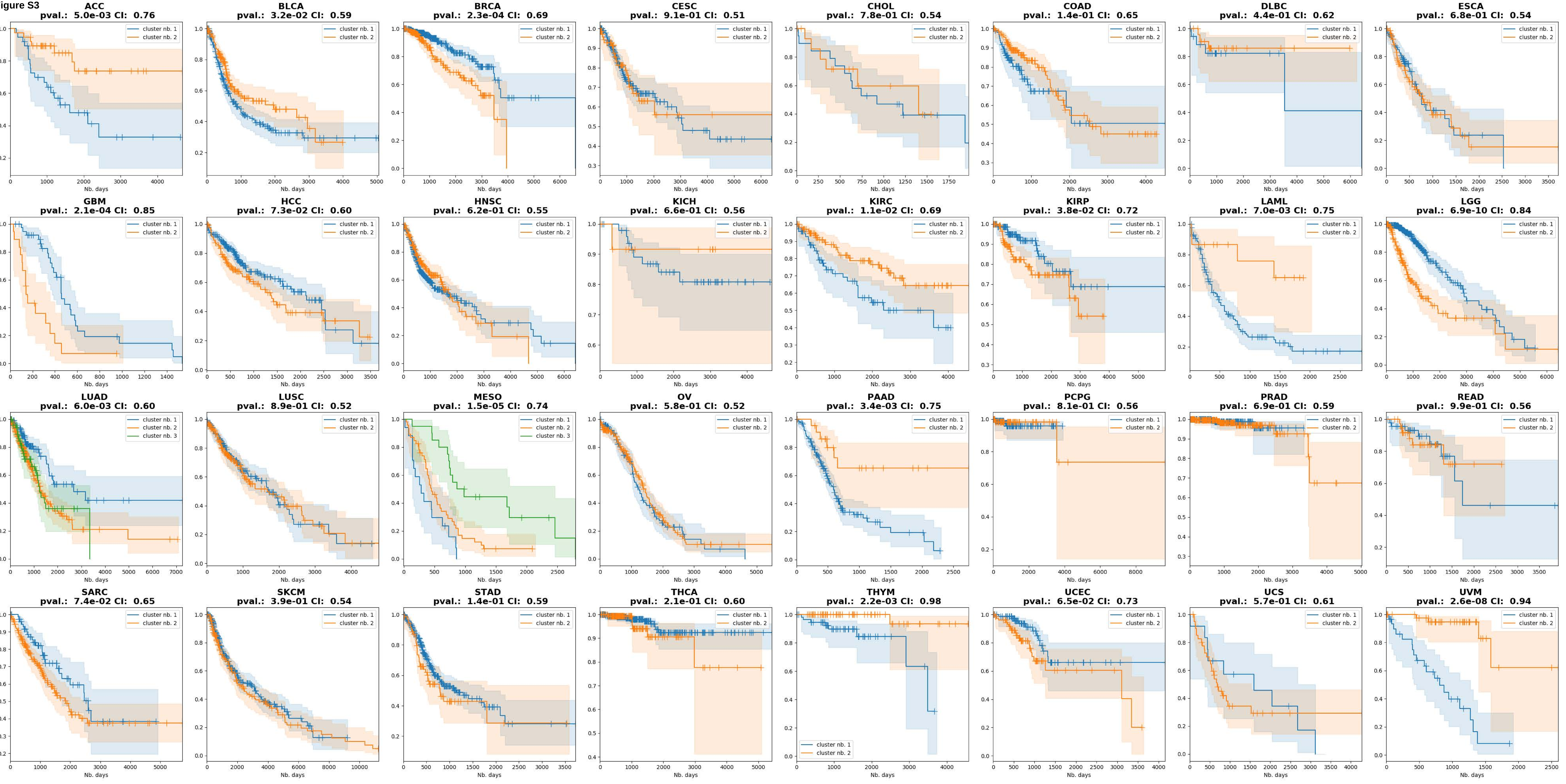
ACC
pval.: 5.0e-03 CI: 0.76
BLCA
pval.: 3.2e-02 CI: 0.59
BRCA
pval.: 2.3e-04 CI: 0.69
CESC
pval.: 9.1e-01 CI: 0.51
CHOL
pval.: 7.8e-01 CI: 0.54
COAD
pval.: 1.4e-01 CI: 0.65
DLBC
pval.: 4.4e-01 CI: 0.62
ESCA
pval.: 6.8e-01 CI: 0.54
GBM
pval.: 2.1e-04 CI: 0.85
HCC
pval.: 7.3e-02 CI: 0.60
HNSC
pval.: 6.2e-01 CI: 0.55
KICH
pval.: 6.6e-01 CI: 0.56
KIRC
pval.: 1.1e-02 CI: 0.69
KIRP
pval.: 3.8e-02 CI: 0.72
LAML
pval.: 7.0e-03 CI: 0.75
LGG
pval.: 6.9e-10 CI: 0.84
LUAD
pval.: 6.0e-03 CI: 0.60
LUSC
pval.: 8.9e-01 CI: 0.52
MESO
pval.: 1.5e-05 CI: 0.74
OV
pval.: 5.8e-01 CI: 0.52
PAAD
pval.: 3.4e-03 CI: 0.75
PCPG
pval.: 8.1e-01 CI: 0.56
PRAD
pval.: 6.9e-01 CI: 0.59
READ
pval.: 9.9e-01 CI: 0.56
SARC
pval.: 7.4e-02 CI: 0.65
SKCM
pval.: 3.9e-01 CI: 0.54
STAD
pval.: 1.4e-01 CI: 0.59
THCA
pval.: 2.1e-01 CI: 0.60
THYM
pval.: 2.2e-03 CI: 0.98
UCEC
pval.: 6.5e-02 CI: 0.73
UCS
pval.: 5.7e-01 CI: 0.61
UVM
pval.: 2.6e-08 CI: 0.94
cluster nb. 1
cluster nb. 2
cluster nb. 3
Nb. days

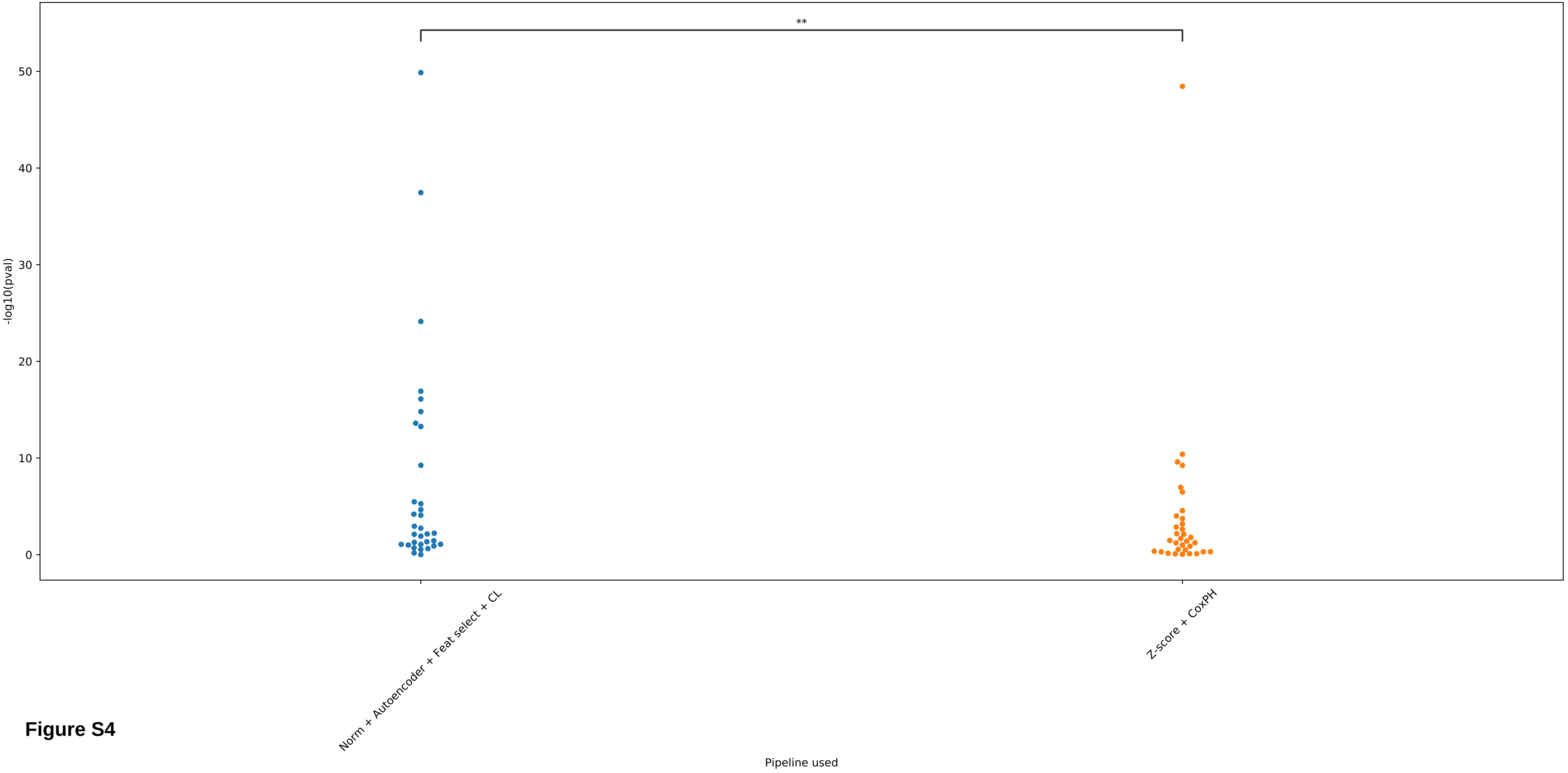
**
50
40
30
20
10
0
-log10(pval)
Norm + Autoencoder + Feat select + CL
Z-score + CoxPH
Pipeline used

Figure S4

Figure S 5

COAD and READ RNA-Seq DeepProg only models

COAD Training

pval.: 3.3e-04 CI:0.64

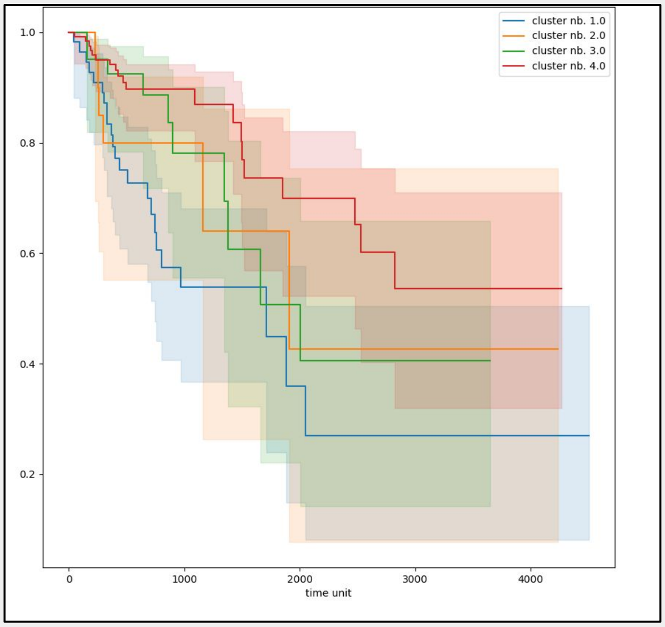

READ test

pval.: 1.4e-02 CI:0.70

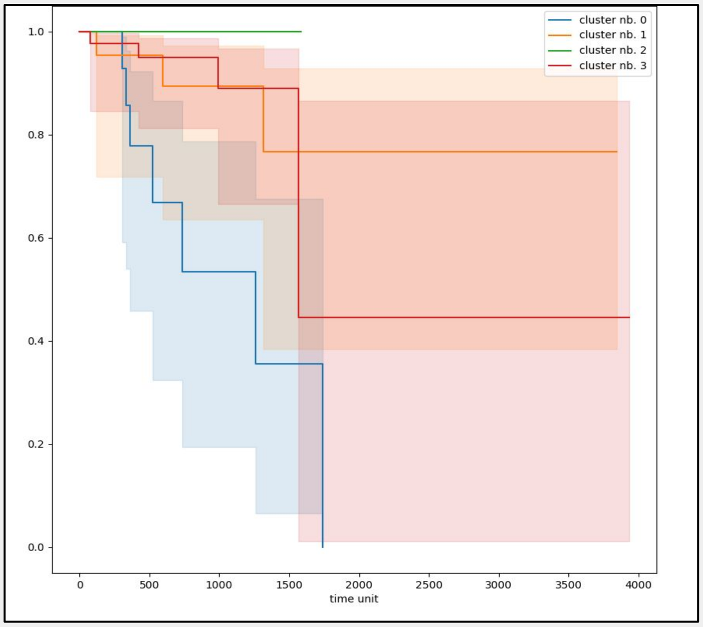

READ training

pval.: 4.3e-03 CI: 0.68

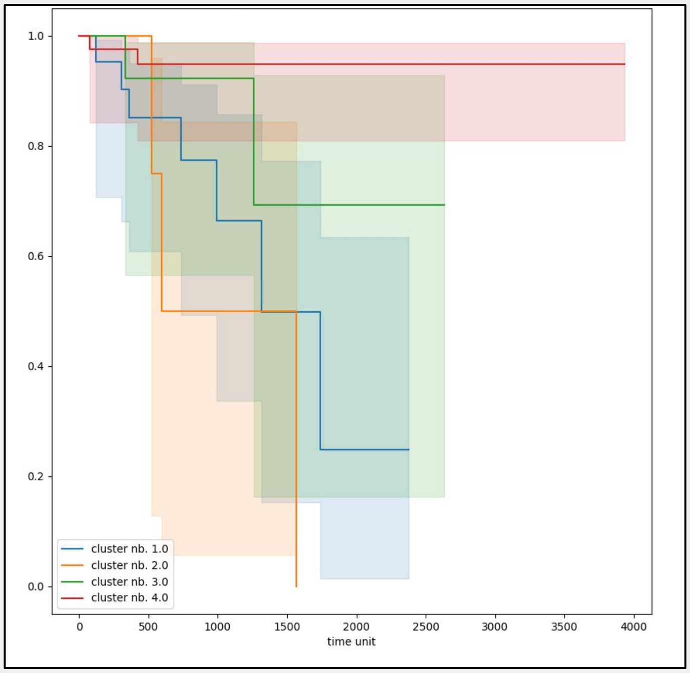

COAD test

pval.: 5.7e-03 CI: 0.62

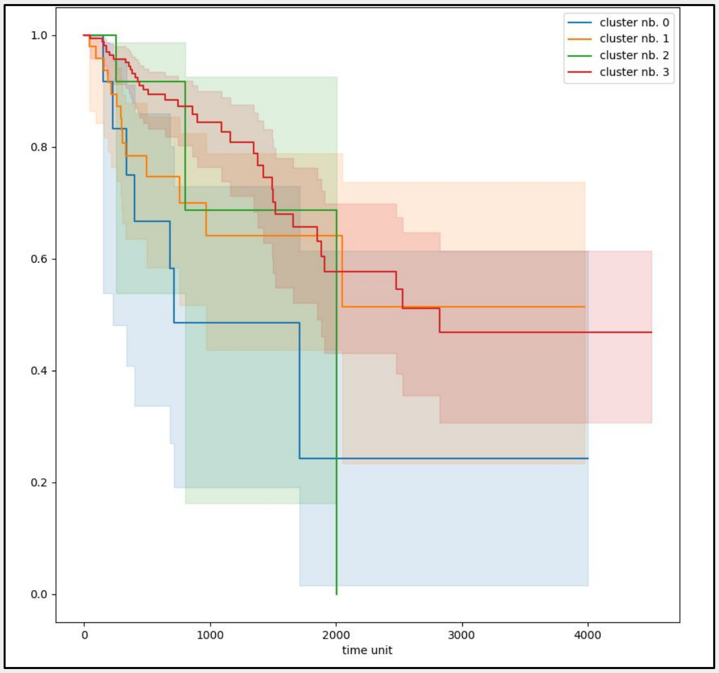

Supplement: Supplementary file 4 — Additional File 4: (Figure S1-S5): Additional information including details of DeepProg model and its comparison with other methods. Figure S1. Autoencoder hidden-layer features significantly associated with survival for each omic data type and cancer type. Figure S2. Compare the performance of DeepProg vs. a simple model based on stage stratification (stage I + II vs. Stage III + IV) on 32 cancer types, on their distributions of the –log10(log-rank p value) of stratified survival curves differences. And compare the performance of DeepProg vs. a simple model based on late stage (Stage III / IV) STAD (B-C) and COAD (D-E): DeepProg survival plots; (B, D): survival plots using stage stratification (C, E). Figure S3. Kaplan-Meier plots for each cancer type, where the survival risk group stratification is determined by SNF, with the same datasets used from the DeepProg analysis in Figure 2A. Figure S4. DeepProg has significantly better (more significant) log-rank p values compared to the baseline Cox-PH model. Figure S5. COAD and READ mutual predictability analysis using RNA as features. A) Subtypes inferred with DeepProg for COAD and used to predict READ subtypes. B) READ subtypes inferred with DeepProg and used to predict COAD subtypes. [file 13073_2021_930_MOESM4_ESM.pdf]
