# Supplementary material for: DeepProg: an ensemble of deep-learning and machine-learning models for prognosis prediction using multi-omics data
Source: Genome Med. 2021 Jul 14;13:112. doi: 10.1186/s13073-021-00930-x (PMC8281595; doi:10.1186/s13073-021-00930-x)
Supplement: Supplementary file 7 — Additional File 7. Additional analysis of microRNA and methylation signatures between the worst survival subtype vs. the remaining samples in each cancer. [file 13073_2021_930_MOESM7_ESM.docx]

## Supplementary information for

**Multi-omics-based pan-cancer prognosis prediction using an ensemble of deep-learning and machine-learning models**

Olivier Poirion^1^, Kumardeep Chaudhary^2^, Sijia Huang^3^, Lana X. Garmire^4*^

**^1^** University of California San Diego, Center for Epigenomics, 9500 Gilman Dr, La Jolla, CA 92093, USA

**^2^** Icahn School of Medicine at Mount Sinai, Department of Genetics and Genomic Sciences, 1 Gustave L. Levy Pl, New York, NY 10029, USA

**^3^** University of Pennsylvania, Department of Biostatistics, Epidemiology and Informatics

Philadelphia, PA, 19104, USA

**^4^** University of Michigan, Department of Computational Medicine and Bioinformatics, Ann Arbor, MI 48105, USA

* Corresponding author.

Emails:

LXG: lgarmire@med.umich.edu

### Prognostic power of the different omics

To rank the relative prognostic importance among RNA-Seq, miRNA, and Methylation data, we next identify the hidden-layer features significantly linked to survival in DeepProg. Since the autoencoder transformed an individual omic matrix into a reduced latent space of 100 new features (**Figure 1**), we report the average number of significant survival features arising from these 100 latent features, for each omic data type and each cancer (**Supplementary Figure 1**). Overall, RNA-Seq has the most important contribution towards survival prediction (average 12.6 /100 significant features), followed closely by miRNA (average 10.8 / 100 features) and then DNA methylation (average 8.3 /100 features). Moreover, RNA-Seq is the most important feature type in 14 out of 32 cancers, compared to miRNA (10 cancers) and Methylation (9 cancers). There is also a wide range of variation for feature importance of each omic data type among cancers. For example, for KICH, the three omics have close to equal level of survival correlations (~5 significant hidden-layer features for each omic), whereas HCC has much different numbers of significant features, with a value of 28 for RNA, 10 for miRNA and 5 for DNA methylations.

### DNA methylation features

Strikingly, we find 15 genes from the Protocadherin Gamma Subfamily (PCDHG) hyper-methylated among the top 100 genes **(Supplementary Table 5)**. This superfamily of cell-adhesion molecules has their genes organized in clusters and hyper-methylation combined with under-expression of protocadherin clusters were described in multiple cancers ^1^. Additionally, xix genes (HOXD13, HOXC5, HOXC4, HOXD10, HOXB4, and HOXA6) from the homeobox (HOX) family are hyper-methylated in the top 100 genes. HOX genes are transcription factors that are involved in normal cell proliferation and development and are also known to be involved with cancer progression ^2^. For example, hyper-methylation of HOXD13 ^3^, HOXC5, and HOXC4 ^4^, were associated with poor prognosis and metastasis progression**.** Interestingly, GPD1 is ranked overall 2nd in our analysis, hyper-methylated in 13 cancers (aggressive subtype) and is associated with survival in 4 cancers. The strong association of GPD1 with tumor progression is new, in that previous literature on this aspect is lacking. The top 2 mostly hypomethylated genes are YOD1 (1^st^), and CYFIP1 (2^nd^). YOD1 is a deubiquitinase that acts as a regulator of the Hippo pathway and a potential therapeutic target to treat liver cancer^5^. Following the literature, YOD1 is significantly associated with survival in the TCGA HCC dataset (log-rank p-value =0.005). CYFIP1 is was shown essential for breast cancer metastasis^6^, and is also significantly associated with survival in the TCGA BRCA dataset (long-rank p-value=0.04).

#### miRNA expression signature

DeepProg pan-cancer analysis detects miR-130b (1^st^), miR-199-a1 (2^nd^) and miR-199-a2 (3^rd^) as the most over-expressed miRNA signatures, and miR-29c and miR101-1 as the most under-expressed miRNA signature (**Supplementary Table 5**). miR-130b over-expression was previously associated with HCC and Ewing sarcoma and metastasis proliferation ^7,8^. Both miR-199-a1 and miR-199-a2 up-regulation were associated with glioblastoma ^9^. On the other hand, miR-29c was shown to suppress metastasis invasion in nasopharyngeal carcinoma ^10^ and HCC ^11^, and miR-101-1 was characterized as a tumor suppressor and metastasis repressor in various cancers ^12,13^.

### References

1. Wang, K.-H. *et al.* Global methylation silencing of clustered proto-cadherin genes in cervical cancer: serving as diagnostic markers comparable to HPV. *Cancer Med.* **4**, 43–55 (2015).

2. Bhatlekar, S., Fields, J. Z. & Boman, B. M. HOX genes and their role in the development of human cancers. *J. Mol. Med.* **92**, 811–823 (2014).

3. Zhong, Z. *et al.* HOXD13 methylation status is a prognostic indicator in breast cancer. *Int. J. Clin. Exp. Pathol.* **8**, 10716–24 (2015).

4. Marzese, D. M. *et al.* Epigenome-wide DNA methylation landscape of melanoma progression to brain metastasis reveals aberrations on homeobox D cluster associated with prognosis. *Hum. Mol. Genet.* **23**, 226–38 (2014).

5. Kim, Y. & Jho, E. Deubiquitinase YOD1: the potent activator of YAP in hepatomegaly and liver cancer. *BMB Rep.* **50**, 281 (2017).

6. Teng, Y. *et al.* The WASF3--NCKAP1--CYFIP1 complex is essential for breast cancer metastasis. *Cancer Res.* **76**, 5133–5142 (2016).

7. Chang, R.-M., Xu, J.-F., Fang, F., Yang, H. & Yang, L.-Y. MicroRNA-130b promotes proliferation and EMT-induced metastasis via PTEN/p-AKT/HIF-1α signaling. *Tumor Biol.* **37**, 10609–10619 (2016).

8. Satterfield, L. *et al.* miR-130b directly targets ARHGAP1 to drive activation of a metastatic CDC42-PAK1-AP1 positive feedback loop in Ewing sarcoma. *Int. J. Cancer* **141**, 2062–2075 (2017).

9. Gu, S. *et al.* Molecular Mechanisms of Regulation and Action of microRNA-199a in Testicular Germ Cell Tumor and Glioblastomas. *PLoS One* **8**, e83980 (2013).

10. Liu, N. *et al.* MiR-29c suppresses invasion and metastasis by targeting TIAM1 in nasopharyngeal carcinoma. *Cancer Lett.* **329**, 181–188 (2013).

11. Lu, Y. *et al.* MiR-29c inhibits cell growth, invasion, and migration of pancreatic cancer by targeting ITGB1. *Onco. Targets. Ther.* **9**, 99–109 (2016).

12. CHO, H. M. *et al.* microRNA-101 inhibits lung cancer invasion through the regulation of enhancer of zeste homolog 2. *Exp. Ther. Med.* **2**, 963–967 (2011).

13. Tang, X.-R. *et al.* MicroRNA-101 inhibits invasion and angiogenesis through targeting ITGA3 and its systemic delivery inhibits lung metastasis in nasopharyngeal carcinoma. *Cell Death Dis.* **8**, e2566–e2566 (2017).
